# Allergen-Specific Treg Cells Upregulated by Lung-Stage *S. japonicum* Infection Alleviates Allergic Airway Inflammation

**DOI:** 10.3389/fcell.2021.678377

**Published:** 2021-06-08

**Authors:** Zhidan Li, Wei Zhang, Fang Luo, Jian Li, Wenbin Yang, Bingkuan Zhu, Qunfeng Wu, Xiaoling Wang, Chengsong Sun, Yuxiang Xie, Bin Xu, Zhaojun Wang, Feng Qian, Jiaxu Chen, Yanmin Wan, Wei Hu

**Affiliations:** ^1^NHC Key Laboratory of Parasite and Vector Biology (National Institute of Parasitic Diseases, Chinese Center for Disease Control and Prevention), Shanghai, China; ^2^State Key Laboratory of Genetic Engineering, Ministry of Education Key Laboratory of Contemporary Anthropology, Human Phenome Institute, Fudan University, Shanghai, China; ^3^Ministry of Education Key Laboratory for Biodiversity Science and Ecological Engineering, Department of Microbiology and Microbial Engineering, School of Life Sciences, Fudan University, Shanghai, China; ^4^Department of Infectious Diseases, Huashan Hospital, Fudan University, Shanghai, China; ^5^Department of Immunology and Microbiology, Shanghai Jiao Tong University School of Medicine, Shanghai, China; ^6^Department of Radiology, Shanghai Public Health Clinical Center, Fudan University, Shanghai, China

**Keywords:** allergic airway inflammation, asthma, helminth therapy, Treg, *Schistosoma* infection, parasite–host interaction

## Abstract

*Schistosoma japonicum* infection showed protective effects against allergic airway inflammation (AAI). However, controversial findings exist especially regarding the timing of the helminth infection and the underlying mechanisms. Most previous studies focused on understanding the preventive effect of *S. japonicum* infection on asthma (infection before allergen sensitization), whereas the protective effects of *S. japonicum* infection (allergen sensitization before infection) on asthma were rarely investigated. In this study, we investigated the protective effects of *S. japonicum* infection on AAI using a mouse model of OVA-induced asthma. To explore how the timing of *S. japonicum* infection influences its protective effect, the mice were percutaneously infected with cercaria of *S. japonicum* at either 1 day (infection at lung-stage during AAI) or 14 days before ovalbumin (OVA) challenge (infection at post–lung-stage during AAI). We found that lung-stage *S. japonicum* infection significantly ameliorated OVA-induced AAI, whereas post–lung-stage infection did not. Mechanistically, lung-stage *S. japonicum* infection significantly upregulated the frequency of regulatory T cells (Treg cells), especially OVA-specific Treg cells, in lung tissue, which negatively correlated with the level of OVA-specific immunoglobulin E (IgE). Depletion of Treg cells *in vivo* partially counteracted the protective effect of lung-stage *S. japonicum* infection on asthma. Furthermore, transcriptomic analysis of lung tissue showed that lung-stage *S. japonicum* infection during AAI shaped the microenvironment to favor Treg induction. In conclusion, our data showed that lung-stage *S. japonicum* infection could relieve OVA-induced asthma in a mouse model. The protective effect was mediated by the upregulated OVA-specific Treg cells, which suppressed IgE production. Our results may facilitate the discovery of a novel therapy for AAI.

## Introduction

The prevalence of asthma has increased dramatically in the past three decades (Eder et al., 2006; Ali, 2011) and represents a great health burden, especially in developed countries (Barnes, 2004; Thomsen, 2015). Atopic asthma is the most common form of asthma. It is an immunological disorder characterized by inflammation of the airways and lungs triggered by allergen with marked T_H_2 responses, overactive immunoglobulin E (IgE) production, mucus hypersecretion, and large amount of eosinophil influx to the airways (Lambrecht and Hammad, 2015).

The exact social and environmental factors that lead to the hyperreactive immune disorder is not fully understood. A leading theory behind the rapid rise of allergy and asthma rates is the “hygiene hypothesis,” which suggests that the decreasing incidence of infections in Western countries may contribute to the rise of both autoimmune and allergic diseases (Okada et al., 2010). This hypothesis was supported by an observation showing that the Western lifestyle was linked with significantly higher prevalence of atopic diseases (Herbert et al., 2009). A putative explanation to this phenomenon is that the overall reduction in common T_H_1-inducing (bacterial, viral, and parasitical) infections results in a decreased ability to counterbalance T_H_2-polarized allergic diseases (Umetsu, 2012; Yang et al., 2016; Stiemsma and Turvey, 2017). Following this lead, a variety of experimental studies have shown that helminth infections can downregulate host immunity and immunopathology in allergy and other immune disorders (Sitcharungsi and Sirivichayakul, 2013; Maizels, 2016; Maizels and McSorley, 2016). Schistosome was one of the parasites that have been found to have protective effects against autoimmune diseases and allergies such as arthritis and asthma (Osada et al., 2009; Janssen et al., 2016; Qiu et al., 2017). These explorations hold great promise in identifying a new and more specific intervention measure for atopic asthma that does not result in certain side effects, such as increased susceptibility to infection and necrosis, which can be triggered by steroid hormone drugs such as dexamethasone (Kuprys-Lipinska and Kuna, 2014; Al-Ahmad et al., 2018; Falk, 2018).

Schistosome is an ancient parasite affecting more than 230 million people in 78 tropical and subtropical countries (LoVerde, 2019). During its life stages in definitive hosts, schistosome invades its mammalian hosts through the skin, migrates from skin to lung, and then develops and matures in the liver, finally residing in the mesenteric venules (McManus et al., 2018). Although it has been shown by multiple studies that schistosome could abate allergic airway inflammation (AAI), the understanding of its underlying mechanisms remains limited. Most previous studies focused on testing the preventive effect (infection before allergen sensitization) of the *Schistosoma japonicum* infection against allergic asthma. Under this setting, controversial results have been reported regarding both the timing of the infection (acute vs. chronic) (Smits et al., 2007; van der Vlugt et al., 2012; Layland et al., 2013) and the effector component (egg vs. worms) (Mangan et al., 2006; Pacifico et al., 2009; Obieglo et al., 2018), which reflects the complexities of the schistosome life cycle and its immune regulatory components. Moreover, contradictory results were also reported regarding the roles of regulatory T cells (Treg cells) in schistosome-mediated protection. Some studies showed that Treg cell was an important effector in schistosome-mediated protection against asthma (Medeiros et al., 2003; Smits et al., 2007; Pacifico et al., 2009; Layland et al., 2013; Zhang et al., 2019), whereas a more recent study showed that the protection was independent of Treg cells (Obieglo et al., 2018).

Unlike previous studies that were focused on testing the preventive effect (infection before allergen sensitization) of *S. japonicum* infection against allergic asthma, the primary goal of this study was to investigate the protective effect of *S. japonicum* infection on asthmatic inflammation (infection after allergen sensitization) and to clarify the underlying mechanism. To this aim, mice were percutaneously infected with cercaria of *S. japonicum* at either 1 day before OVA-induced asthma attack (infection at lung-stage during AAI) or 14 days before OVA-induced asthma attack (infection at post–lung-stage during AAI). We found that only lung-stage *S. japonicum* infection could upregulate the frequency of allergen-specific Treg cell, which significantly alleviated AAI by inhibiting IgE production and inflammatory cytokine secretion.

## Materials and Methods

### Ethics Statement

All experiments and methods were performed in accordance with relevant guidelines and regulations. Mice experiments were carried out at the National Institute of Parasitic Disease, Chinese Center for Disease Control and Prevention (NIPD, China CDC) in Shanghai, China. All animal experiment protocols used in this study were approved by the Laboratory Animal Welfare & Ethic Committee of the National Institute of Parasitic Diseases (permit no. IPD-2016-7).

### OVA-Induced AAI and *S. japonicum* Infection

**FIGURE 1:**
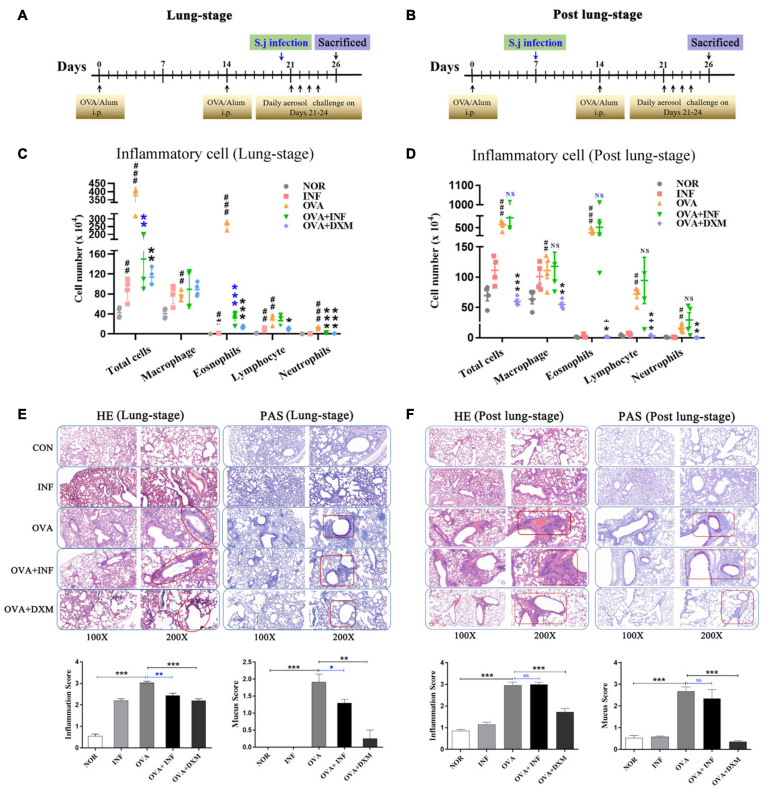
Lung-stage *S. japonicum* infection alleviated the attack of OVA-induced AAI, whereas post–lung-stage infection did not. Experimental design of OVA-induced AAI treated with either lung-stage **(A)** or post–lung-stage **(B)** *S. japonicum* infection in murine model. **(C,D)** Inflammatory cell (total cell, macrophage, eosnophils, lymphocytes, neutrophils) infiltration in BALF of mice after OVA challenge was compared in NOR, INF, OVA, OVA + INF, and OVA + DXM groups (*n* = 5 or 6 mice per group, experiment performed twice). **(E,F)** Representative images of H&E and PAS staining of lung tissue after OVA challenge. Statistical analysis of inflammation score and mucus secretion score are also shown in **(E,F)**, respectively, among five groups (*n* = 5 or 6 mice per group, experiment performed twice). NOR, normal mice (without OVA sensitization and challenge); INF, mice without OVA sensitization and challenge but infected with schistosome; OVA, mice with OVA sensitization and challenge but without *S. japonicum* infection; OVA + INF, mice sensitized and challenged with OVA and treated with *S. japonicum* infection; OVA + DXM, mice sensitized and challenged with OVA and treated with dexamethasone. All data are shown as mean ± SEM. *P < 0.05, **P < 0.01; NS, not significant by one-way analysis of variance (ANOVA) with Tukey test. #, ##, and ### indicate *P* < 0.05, <0.01, and <0.001, respectively, OVA versus NOR **(C,D)**. *, **, *** indicated *P* < 0.05, <0.01, and <0.001, respectively, OVA + INF or OVA + DXM versus OVA **(C,D)**.

Female BALB/c mice (6–8 weeks old) were randomly divided into six groups in this experiment, which were OVA-induced AAI (OVA) group, OVA-induced AAI with lung-stage *S. japonicum* infection (OVA + INF, lung stage) group, OVA-induced AAI with post–lung-stage infection (OVA + INF, post–lung stage) group, and OVA-induced AAI with dexamethasone (DXM) treatment (OVA + DXM) group, as well as infection (INF) group and normal (NOR) group. For OVA-induced AAI, the mice were sensitized by injecting 10 μg of alum-adjuvanted ovalbumin (OVA; cat. # 77120 and 77161; Thermo Fisher, United States) intraperitoneally on days 0 and 14. Subsequently, the mice were challenged with aerosolized OVA [1% in phosphate-buffered solution (PBS)] for 30 min in the chamber of a Medical Compressor Nebulizer (DEDAKJ, Germany) on days 21 to 24 (Figures 1A,B). The mice of the normal control and *S. japonicum* infection control groups were challenged with PBS. To test the protective effect of infection on OVA-induced AAI, mice were infected with 15 cercaria of *S. japonicum* at either 1 day (infection within 6 days: lung-stage infection) or 14 days before OVA-induced asthma attack (20 days within infection: post–lung-stage infection).

### Bronchoalveolar Lavage Collection and Cell Counting

Mice were euthanized 48 h after the last aerosolized OVA challenge (day 26), and bronchoalveolar lavage fluids (BALFs) were collected as previously reported method (Li et al., 2012). Briefly, after euthanasia, tracheotomy was carried out, and an arteriovenous indwelling needle (20-gauge; BRAUN, Germany) was inserted into the trachea. Lavages were collected by washing the lung twice with 0.3 mL PBS. Cells in BALFs were harvested after centrifugation, and the supernatants were stored at –80°C for cytokine detection. Cell pellet was fixed with paraformaldehyde (4%) and stained with a hematoxylin–eosin (H&E). A total of 1,000 cells from multiple fields were examined for each slide. Counts of total cells, eosinophils, macrophage, neutrophils, and lymphocytes were performed on blinded samples, as described previously (Chang and Yen, 2004).

### Lung Histopathology

Lung tissues were fixed in 4% phosphate-buffered formaldehyde overnight and then embedded in paraffin and cut for H&E and periodic acid–Schiff (PAS) staining. Images of the stained sections were captured with a NIKON DS-U3 microscope (NIKON, Japan). Lung inflammation and the intensity of goblet cell metaplasia were assessed and scored 0 to 4 by two blinded, independent investigators, as described previously (Hopfenspirger and Agrawal, 2002).

### Determination of Total and OVA-Specific IgE in Serum

The levels of total and OVA-specific IgE in serum were measured using enzyme-linked immunosorbent assay. Briefly, Maxisorp 96-well microtiter plates (Thermo Fisher Scientific, United States) were coated with rat monoclonal anti-mouse IgE antibody for total IgE detection (1:1,000; cat. # ab99571, Abcam, United Kingdom) or 10 μg/mL OVA for OVA-specific IgE (cat. # A5503, Sigma, United States) 100 μL/well, respectively, in carbonate–bicarbonate buffer, pH 9.6, for 12–16 h at 4°C. Then, the plates were blocked for at least 2 h at 37°C with 100 μL/well of PBS plus bovine serum albumin (BSA) (1%). After wash, 100 μL serum diluted with PBS containing 0.05% Tween 20 (PBST) (1:40 for total IgE; 1:5 for OVA-specific IgE) was added to each well and incubated at 37°C for 2 h. Next, horseradish peroxidase–labeled goat anti-mouse IgE antibody was diluted with PBST (1:2,000; cat. # ab99574, Abcam, United Kingdom) and added to each well at 100 μL/well. After 2 h of incubation at 37°C, the plates were washed with PBST five times. Finally, color was developed by addition of 100 μL/well of TMB (cat. # PA107, TIANGEN, China), and after incubation at room temperature for maximal 30 min, the reaction was stopped with 5% sulfuric acid (50 μL/well). Optical density values were determined at 450 nm using the multimode microplate readers (BioTek, United States). The concentration of total IgE was then calculated according to the standard curve.

### Cytokine Detection in BALFs

Levels of interleukin 4 (IL-4), IL-5, IL-13, IL-10, eotaxin, and interferon γ (IFN-γ) in BALFs were measured using a custom-made Bio-Plex Pro Reagent Kit V (6-plex customization) (cat. # MHSTCMAG-70K, Wayen Biotechnologies, China) according to the manufacturer’s instructions. The fluorescence-labeled beads were detected using a corrected Bio-Plex MAGPIX system (Bio-Rad, Luminex Corporation, Austin, TX, United States), and the cytokine concentrations were calculated using Bio-Plex manager 6.1 (Bio-Rad).

### Lymphocyte Isolation From Lung Tissues

After collection, lung tissues were washed three to four times with RPMI (Roswell Park Memorial Institute) medium, minced to tiny pieces, and then digested in 0.1% type IV collagenase (cat. # C8160, Solarbio, China) solution at 37°C for 30 min. Digested lung tissues were filtered through a 70-μm cell strainer, and erythrocytes were lysed with a red blood cell lysis buffer (cat. # R1010, Solarbio, China).

### Flow Cytometry Assay

Single-cell suspensions were stained with a panel of surface monoclonal antibodies (mAbs) in FACS buffer (PBS containing 2 mM EDTA and 0.5% BSA) for 30 min on ice, including fluorescein isothiocyanate (FITC)–conjugated anti-CD4 (clone # 88-8111-40, eBioscience, United States), APC-conjugated anti-CD25 mAb (clone # 88-8111-40, eBioscience, United States), SuperBright645-conjugated anti-CD45.1 (clone # 64-0453-82, eBioscience, United States), and Pe-cyanine7–conjugated anti-CD45.2 (clone # 25-0453-82, eBioscience, United States). Subsequently, cells were fixed with fix/perm buffer (clone # 88-8111-40, eBioscience, United States) on ice for 20 min, and then stained with mAbs targeting intracellular markers in a Perm/wash buffer for 30 min on ice. For the detection of Treg cell, PE-labeled anti-Foxp3 mAb (clone # 88-8111-40, eBioscience, United States) was used. For detecting OVA-specific IL-4 and IFN-γ secretion, isolated lymphocytes were initially stimulated for 6 h with 5 μg/mL OVA peptide (323-339) (China peptides, China); leukocyte activation cocktail, with BD GolgiPlug (BD, United States) was added for another 4 h and then stained with mAbs Perp-cy5.5–conjugated anti-CD3 (clone # 145-2C11, eBioscience, United States) and FITC-conjugated anti-CD4 (clone # 88-8111-40, eBioscience, United States) for 30 min on ice. Subsequently, cells were fixed with fix/perm buffer (clone # 88-8111-40, eBioscience, United States) on ice for 20 min. Then PE-conjugated anti–IL-4 (clone # 12-7041-81, eBioscience, United States) or APC-conjugated anti-IFN-γ (clone # 17-7311-81, eBioscience, United States) for 30 min on ice was used. Finally, after two washes, all cells were resuspended in PBS containing 1% paraformaldehyde and subjected to flow cytometry analysis (Cytometer LX, Beckman).

### Adoptive Transfer of Naive CD4^+^ T Cells

Naive CD4^+^ T cells of CD45.1^+^ OT II mice were purified using EasySep Mouse Naive CD4^+^ T Cell Isolation Kit (cat. # 19765, StemCell, United States) according to the manufacturer’s protocol. The purity of isolated cells was checked by flow cytometry and was confirmed to be >85%. Freshly purified naive CD4^+^ T cells were suspended in PBS and injected intravenously into CD45.2^+^ congenic C57BL/6 recipient mice, 1 × 10^6^ cells/mouse. The induction of AAI and *S. japonicum* infection was performed as described above.

### *In vivo* Depletion of Treg Cells

Anti-CD25 antibody clone PC61 has been widely used to deplete Treg cells for characterizing Treg cell function *in vivo* (Setiady et al., 2010); 100 μg/mouse anti-CD25 antibody (cat. # 16-0251-85, clone # PC61.5, eBioscience, United States) or isotype IgG (cat. # 16-4301-85, clone # eBRG1, eBioscience, United States) was dissolved with 150 μL sterile PBS and injected intravenously into the mice 21 days after OVA sensitization. A second shot of 50 μg/mouse antibodies was given on day 23 after OVA sensitization (Figure 7A). After depletion, the mice were randomly divided into two groups: OVA + INF + αCD25 and OVA + INF + IgG. OVA sensitization, aerosol challenge, and *S. japonicum* infection were performed as described above.

### RNA Sequencing

Total RNA was extracted from lung tissues by using Trizol reagent (cat. # 15596026, Invitrogen). RNA purity was checked using the Nano Photometer spectrophotometer (IMPLEN, CA, United States). RNA integrity was assessed using the RNA Nano 6000 Assay Kit of the Bioanalyzer 2100 system (Agilent Technologies, CA, United States); 1 μg total RNA from each sample was used to construct the sequencing library using Poly(A) mRNA Capture Module (cat. # RK20340, Abclonal, United States) and Fast RNA-seq Lib Prep Module for Illumina (cat. # RK20304, Abclonal, United States). Index codes were added to attribute sequences of each sample. Then, the libraries were sequenced on Illumina Novaseq platform [2 × 150 base pairs (bp)]. A total of seven samples, three from the OVA group and four from the OVA + INF group, were sequenced in one lane, producing more than 30 million reads per library.

### Differential Expression Genes Analysis and Functional Enrichment Analysis

Sequencing quality was evaluated by FastQC software^1^. Poor-quality reads and adaptors were trimmed by Trimmomatic software (released version 0.22^2^), and only reads longer than 50 bp were used for further analysis. The high-quality reads were mapped to mouse genome (mouse BALB/cJ) downloaded in Ensembl database. The HTseq (Anders et al., 2015) was used to quantify gene expression, and R DEseq2 package (Anders and Huber, 2010) was employed for differential expression analysis. Only genes with false discovery rate (FDR) adjusted *P* < 0.05 and absolute value of fold change > 2 were considered as differential expression genes (DEGs). Functional enrichment of Gene Ontology (GO) terms and Kyoto Encyclopedia of Genes and Genomes analyses of DEGs were conducted by R Cluster Profiler package (Yu et al., 2012) with FDR correction. Significantly enriched GO terms and KEGG pathways were identified with corrected *P* < 0.05. DEG-related pathways enrichment terms were performed with the Panther Classification System^3^.

Data and materials availability: RNA sequencing data are deposited in the SRA database SRA (SRA accession no. PRJNA609083).

### Statistical Analysis

All statistical analyses were performed using GraphPad Prism 8.0 (GraphPad Software, Inc., San Diego, CA, United States). The data of quantitative variables were presented as mean ± standard error of mean (SEM). *P* < 0.05 was considered statistically significant.

## Results

### Lung-Stage *S. japonicum* Infection Ameliorated OVA-Induced AAI in a Murine Model

A mouse model of OVA-induced AAI was adopted to test the protective effect of *S. japonicum* infection on allergic asthma (Figures 1A,B). Compared to the control group, mice in the OVA group showed significant infiltration of inflammatory cells in BALFs (Figures 1C,D), which resembled the main clinical feature of AAI (Persson, 2019). Moreover, after *S. japonicum* infection, the results showed that the lung-stage infection significantly reduced the infiltration of inflammatory cells, especially eosinophils (Figure 1C), whereas post–lung-stage infection did not (Figure 1D). Histopathologic examination further confirmed the above findings by showing that lung-stage infection significantly suppressed the OVA-induced eosinophil-rich leukocyte infiltration and mucus hypersecretion (Figure 1E), whereas post–lung-stage infection showed no obvious protective effect (Figure 1F).

### Lung-Stage *S. japonicum* Infection Inhibited IgE Production and Suppressed T_H_2 Cytokine Secretions

**FIGURE 2:**
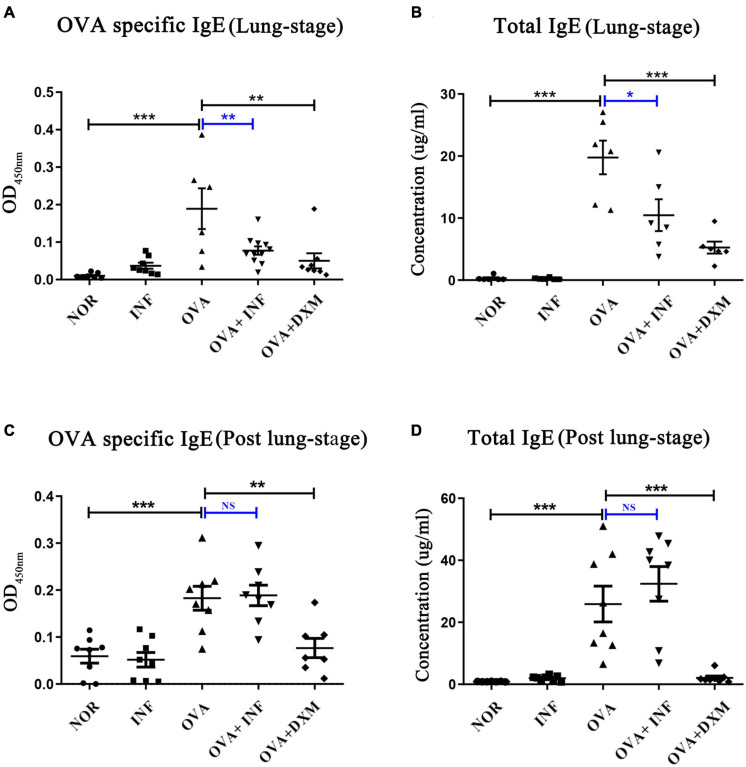
Lung-stage *S. japonicum* infection suppressed both the total and OVA-specific IgE after OVA challenge, whereas post–lung-stage infection did not. **(A,C)** OVA-specific IgE in sera were measured by enzyme-linked immunosorbent assay from NOR, INF, OVA, OVA + INF, and OVA + DXM groups (*n* = 5 or 6 mice per group, experiment performed twice). **(B,D)** The concentrations of total IgE in mouse serum were compared among all groups after OVA challenge (*n* = 5 or 6 mice per group, experiment performed twice). All data are shown as mean ± SEM. *P < 0.05, **P < 0.01, ***P < 0.001; NS, not significant by one-way analysis of variance (ANOVA) with Tukey test.

**FIGURE 3:**
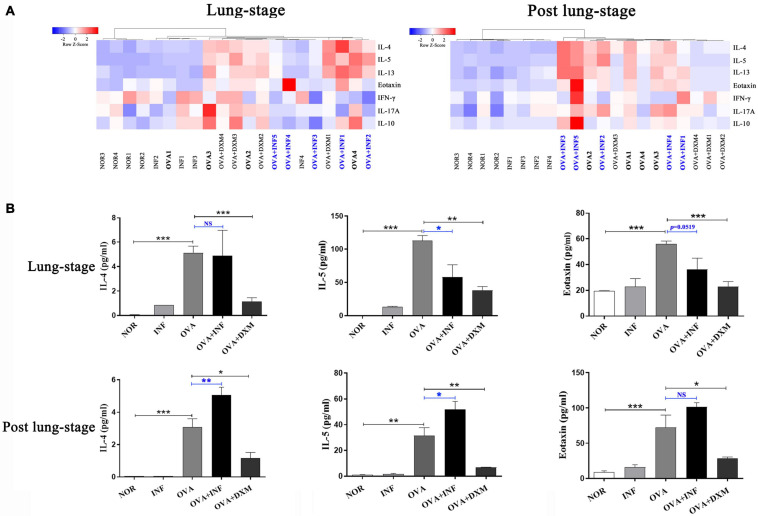
Lung-stage *S. japonicum* infection inhibited T_H_2 cytokine secretion after OVA challenge, while post–lung-stage infection did not. **(A)** Heatmaps of multiple cytokines in BALF of mice treated with lung-stage *S. japonicum* infection (left) and post–lung-stage *S. japonicum* infection (right) after OVA challenge by Luminex (*n* = 5 or 6 mice per group, experiment performed twice). **(B)** Concentrations of IL-4, IL-5 and eotaxin in BALFs were compared among all groups (*n* = 5 or 6 mice per group, experiment performed twice). Data are shown as mean ± SEM. *P < 0.05, **P < 0.01, ***P < 0.001; NS, not significant by one-way analysis of variance (ANOVA) with Tukey test.

IgE is the key factor mediating the pathological immune responses that lead to allergic asthma (Galli and Tsai, 2012). To further characterize the protective effects of *S. japonicum* infection, we measured the total and OVA-specific IgE in serum of mice. The results showed that lung-stage infection significantly downregulated both the total and OVA-specific IgE to levels comparable with DXM treated mice (Figures 2A,B). In contrast, post–lung-stage infection tended to elevate the total and OVA-specific IgE levels despite no significant difference was reached (Figures 2C,D). Moreover, we also measured a panel of cytokines and chemokines in BALFs and found that lung-stage infection altered the cytokine/chemokine secretion pattern induced by aerosolized OVA challenge (Figure 3A and Supplementary Figure 1). More specifically, IL-5 and eotaxin were reduced to levels that are similarly attained with DXM treatment (Figure 3B). On the contrary, post–lung-stage infection increased IL-4 and IL-5 secretion (Figure 3B).

### Lung-Stage *S. japonicum* Infection Upregulated the Frequencies of Treg Cells Especially OVA-Specific Treg Cells in Lung

**FIGURE 4:**
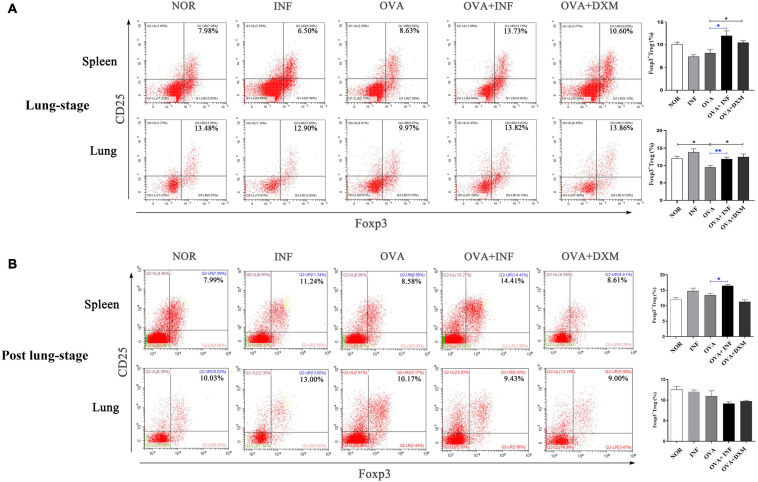
Lung-stage *S. japonicum* infection upregulated Treg cell frequency in lung and spleen after OVA challenge. **(A,B)** Comparisons of Treg cell populations (CD4^+^CD25^+^Foxp3^+^ Treg cell) in lungs and spleens among all groups. Representative data of flow cytometry analysis for each group are shown together with statistical comparisons. *n* = 5 or 6 mice per group, experiment performed twice. *P < 0.05, **P < 0.01 by one-way analysis of variance (ANOVA) with Tukey test.

**FIGURE 5:**
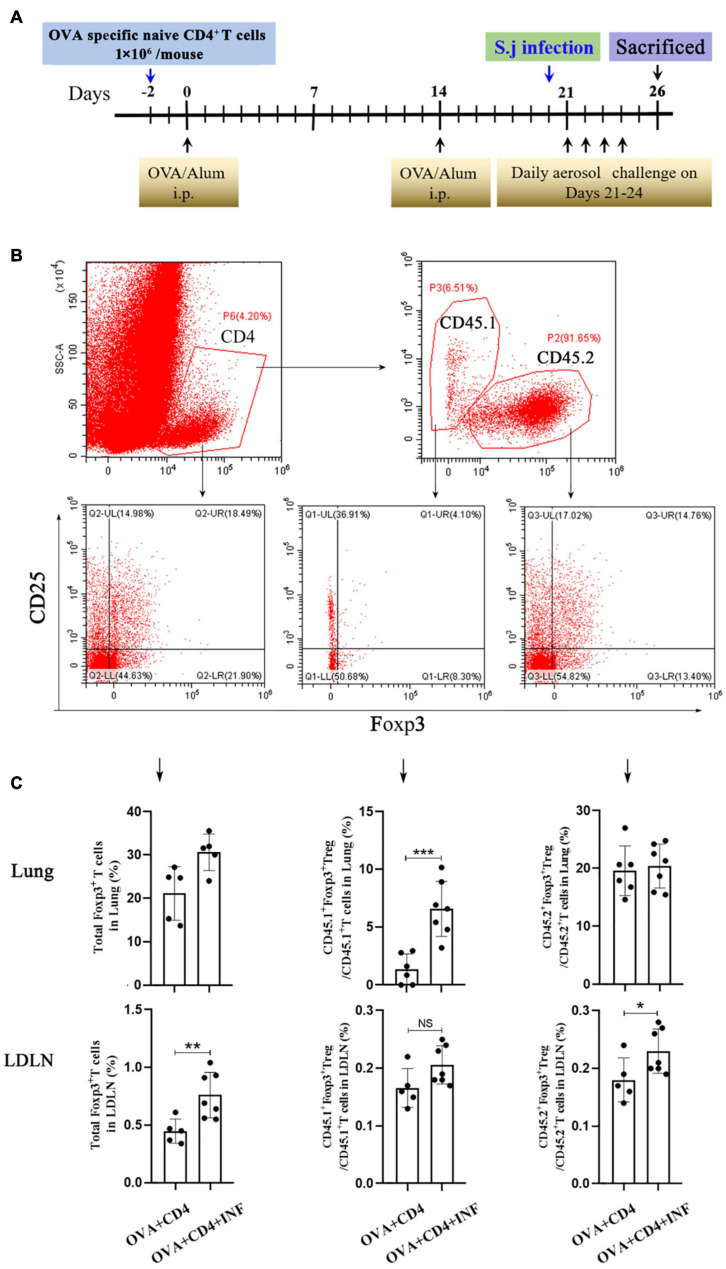
Lung-stage *S. japonicum* infection upregulated OVA-specific Treg cells after OVA challenge. **(A)** Design of experiment for testing the protective effect of lung-stage *S. japonicum* infection on OVA-induced AAI after adoptive transfer of OVA-specific naive CD4^+^ T cells. **(B,C)** Gate strategy and statistical comparisons of flow cytometry analysis for total Treg, CD45.1^+^ Treg cells (OVA-specific) and CD45.2^+^ Treg cells in lung and lung draining lymph nodes (LDLN). *n* = 5 or 6 mice per group, experiment performed twice. *P < 0.05, **P < 0.01, ***P < 0.001; NS, not significant by the one-way analysis of variance (ANOVA) with Tukey test.

Treg cell was suggested to be a key factor of *S. mansoni*–mediated protection against AAI (Layland et al., 2013). Here, we first assessed the frequencies of total Treg cells (CD4^+^CD25^+^Foxp3^+^ Treg cell) in spleen and lung. As shown in Figure 4A, lung-stage infection upregulated the frequency of total Treg cells both in lung and spleen (Figure 4A), whereas post–lung-stage infection only slightly improved the proportion of Treg cells in the spleen (Figure 4B). Then, by adoptive transfer of OVA-specific naive CD4^+^ T cells (CD45.1^+^) into wide-type CD45.2^+^ mice (Figure 5A), we found that the frequency of OVA-specific Treg cells (CD45.1^+^ Treg) in lung increased by more than threefold after *S. japonicum* infection (*P* < 0.001), whereas the frequency of endogenous Treg cells (CD45.2^+^ Treg cell) in lung was not significantly improved (Figures 5B,C). The proportion of total Treg cells was increased in lung and LDLNs after *S. japonicum* infection (Figures 5B,C).

We also found that the ratio of OVA-specific IL-4^+^ versus IFN-γ^+^ CD4^+^ T cells significantly decreased after lung-stage *S. japonicum* infection (Supplementary Figure 2), suggesting that specific CD4^+^ T cell responses shifted from T_H_2 toward T_H_1 responses.

### The Protective Effect of Lung-Stage *S. japonicum* Infection Was Treg Cell–Dependent

**FIGURE 6:**
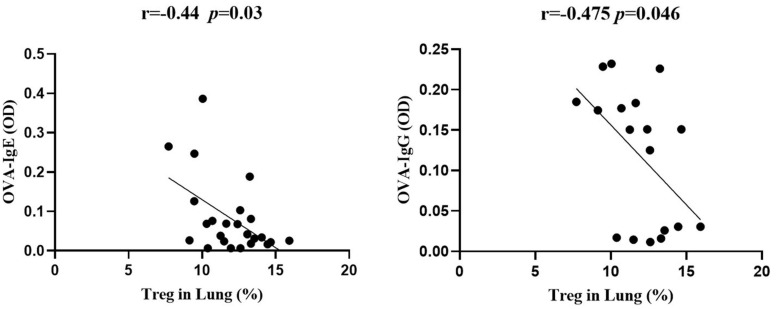
The frequency of Treg cells in lung negatively correlated with OVA-specific IgE and IgG. Correlation analysis between Treg cell frequency in lung and the optical density values of OVA-specific IgE **(left)** and IgG **(right)** in serum (*n* = 5 or 6 mice per group, experiment performed twice).

**FIGURE 7:**
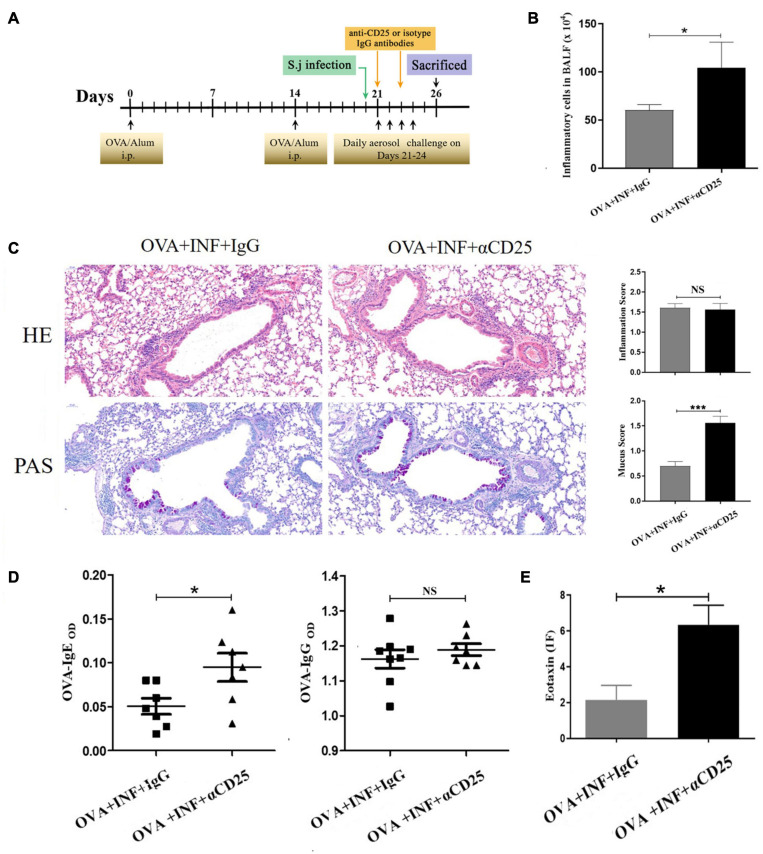
*In vivo* depletion of Treg cells counteracted the protective effect of lung-stage *S. japonicum* infection on OVA-induced AAI. **(A)** Design of experiment for testing the role of Treg cells in the protective effect mediated by lung-stage *S. japonicum* infection. **(B)** Comparisons of total inflammatory cell counts in BALF between lung-stage schistosome-infected mice treated with either anti-CD25 antibody or isotype control IgG (*n* = 8 mice per group, experiment performed twice). **(C)** Lung histopathology analysis of lung-stage schistosome–infected mice treated with either anti-CD25 antibody or isotype control IgG. Upper, H&E staining; lower, PAS staining (*n* = 8 mice per group, experiment performed twice). **(D)** Comparisons of OVA-specific IgE and IgG in sera between Treg cell–depleted and control mice (*n* = 8 mice per group, experiment performed twice). **(E)** Comparisons of eotaxin levels in BALF between lung stage–infected mice treated with either anti-CD25 antibody or isotype control (*n* = 8 mice per group, experiment performed twice). Data are shown as mean ± SEM. *P < 0.05, ***P < 0.001 by one-way analysis of variance (ANOVA) with Tukey test.

Significant negative correlations between the frequency of Treg cells and OVA-specific IgE or IgG (Figure 6) were observed, indicating that the protective effect of *S. japonicum* infection on AAI might be mediated by Treg cell. To elucidate the role of Treg cell, we performed *in vivo* depletion of Treg cells using anti-mouse CD25 antibody (Figure 7A). The efficiency of Treg cell deletion is demonstrated by the significantly reduced percentage of CD25^+^ Treg cells as shown in Supplementary Figure 3. Our data showed that Treg depletion (OVA + INF + αCD25 group) aggravated OVA-induced AAI compared to isotype control group. Inflammatory cell infiltration, mucus secretion (shown by PAS staining), OVA-specific IgE production, and eotaxin secretion significantly increased after Treg depletion (Figure 7).

### Lung-Stage *S. japonicum* Infection Molded the Microenvironment to Facilitate the Generation of Treg Cells

**FIGURE 8:**
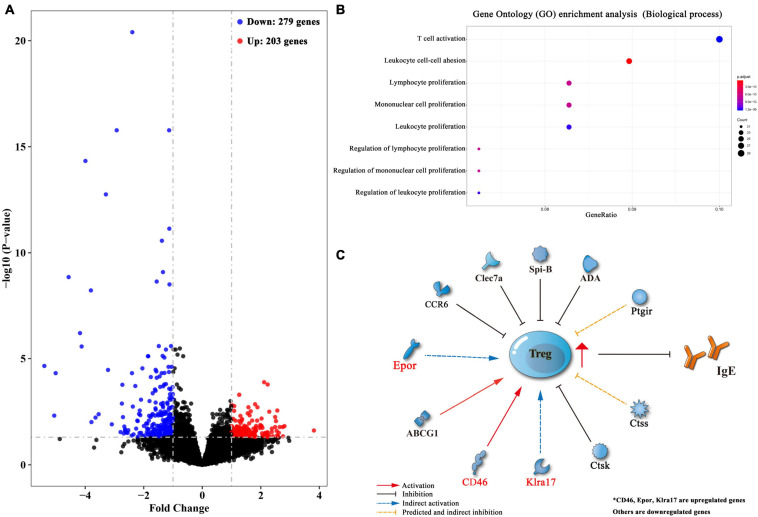
Transcriptomic analysis of differentially expressed genes (DEGs) between lung tissues of OVA-induced asthmatic mice treated with and without lung-stage *S. japonicum* infection. **(A)** Volcano plot of detected gene transcription profile in lung tissues of OVA-induced asthmatic mice treated with lung-stage schistosome infection compared with no-treatment control mice after OVA challenge. **(B)** The top eight functional enrichment pathways of Gene Ontology (GO) analysis for biological process in DEGs (*P* < 0.05). **(C)** Predicted gene network that might promote the generation of Treg cells in DEGs. Data were from at least three individuals per group per experiment, experiment performed twice.

To reveal factors that contributed to the induction of Treg cells upon lung-stage *S. japonicum* infection, we created transcriptomic profiles of the lung tissues from the schistosome infected and non-infected mice after OVA challenge. The results showed that 203 genes were upregulated, and 279 genes were downregulated after lung-stage *S. japonicum* infection (Figure 8A and Supplementary Data File 1). GO analysis of DEGs showed that the top three terms of significantly enriched genes (*P* < 0.05) are mainly distributed in the T cell activation, the leukocyte proliferation, and the regulation of leukocyte proliferation (Figure 8B) pathways. Panther analysis showed that 84 DEGs are related to immune system processes (Supplementary Figure 4), and 70 of them were downregulated (Supplementary Data File 1). Further analysis showed that three genes (CD46, Epor, and Klra17) reported to promote Treg cell response were upregulated (Gehrie et al., 2011; Tsai et al., 2012; Purroy et al., 2017), and eight genes (Clec7a, CCR6, Spi-B, ABCG1, ADA, Ctsk, Ctss, and Ptgir) reported to inhibit Treg cell response were downregulated (Liu et al., 2013; Tang et al., 2015; Cheng et al., 2016; Naval-Macabuhay et al., 2016; Rauch et al., 2016; Yan et al., 2017; Zhou et al., 2017; Kulkarni et al., 2018; Figure 8C and Table 1) in schistosome-infected mice. We postulated that lung-stage *S. japonicum* infection generated a microenvironment facilitating Treg cell development in lung (Figure 8C).

**TABLE 1:** Differential expression genes reported to promote or inhibit Treg cell response.

Classification	Name	Short name	GeneID	Log_2_Foldchange	Adjusted *p*-value	Reference
	CD46 antigen, complement regulatory protein	CD46	17221	1.52	0.015635106	Tsai et al., 2012
Upregulate and promote Treg cell	Erythropoietin receptor	EPOr	13857	1.26	0.000502759	Purroy et al., 2017
	Killer cell lectin-like receptor, subfamily A, member 17	Klra17	170733	1.81	0.00139324	Gehrie et al., 2011
	Chemokine (C-C motif) receptor 6	CCR6	12458	–1.82	0.004889779	Kulkarni et al., 2018
	C-type lectin domain family 7, member a	Clec7a	56644	–1.27	0.016544002	Tang et al., 2015
	Spi-B transcription factor	Spi-B	272382	–1.03	0.008667091	Rauch et al., 2016
Downregulate and inhibit Treg cell	Adenosine deaminase	ADA	11486	–1.45	6.94E-05	Naval-Macabuhay et al., 2016
	ATP binding cassette subfamily G member 1	ABCG1	11307	–1.11	0.024201953	Cheng et al., 2016
	Cathepsin K	Ctsk	13038	–1.30	0.000166352	Zhou et al., 2017
	Cathepsin S	Ctss	13040	–1.44	0.000215072	Yan et al., 2017
	Prostaglandin I receptor	Ptgir	19222	–1.05	0.007454223	Liu et al., 2013
	Programmed cell death 1 ligand 2	Pdcd1lg2	58205	–1.85	0.020944852	Keir et al., 2008
	Interleukin 2 receptor, beta chain	IL-2Rβ	16185	–1.31	0.02093114	Yu et al., 2009
Downregulate and promote Treg cell	CD 5 antigen	CD5	12507	–1.06	0.035912699	Henderson and Hawiger, 2015
	CD52 antigen	CD 52	23833	–1.19	0.001160938	Watanabe et al., 2006
	C-type lectin domain family 4, member a2	DCIR	26888	–1.34	0.001202901	Massoud et al., 2014
	Lipocalin 2	LCN2	16819	–1.57	4.03219E-05	Kudo-Saito et al., 2013

In addition, we found that eight genes (DOCK2, IRF4, Rac2, Lgals3, H2-Oa, Pdcd1lg2, Sash3, and Mzb1) related to B cell function or differentiation (Croker et al., 2002; Scheikl et al., 2009; Flach et al., 2010; Gu et al., 2013; Peng and Eckhardt, 2013; de Oliveira et al., 2018; Jing et al., 2019; Low et al., 2019) were also downregulated after *S. japonicum* infection (Table 2), which might potentially contribute to the inhibition of IgE response. Genes related to lung development (FOXF1, ANO9, TRIM6, MMP27, Epor, Gata1, and Serpina) (Nuttall et al., 2004; Rock et al., 2008; Sato et al., 2012) and cell integrity (Villin and CRB1) (Mehalow et al., 2003; Khurana and George, 2008) were also found to be upregulated too, which indirectly supported the observed protective effect of *S. japonicum* infection (Table 2).

**TABLE 2:** Differential expression genes reported to facilitate B cell or plasma cell, lung development, and cellular morphology.

Classification	Name	Short name	GeneID	Log_2_Foldchange	Adjusted *p*-value	Reference
	Dedicator of cytokinesis	DOCK2	94176	–1.059783027	0.013672224	Jing et al., 2019
	Interferon regulatory factor 4	IRF4	16364	–1.399178091	0.020943206	Low et al., 2019
	Rac family small GTPase 2	Rac2	19354	–1.073471112	0.019595199	Croker et al., 2002
Related to inhibiting IgE production	Lectin, galactose binding, soluble 3	Lgals3	16854	–1.240144306	3.75E-06	de Oliveira et al., 2018
	Histocompatibility 2, O region alpha locus	H2-Oa	15001	–1.349647201	0.001621659	Gu et al., 2013
	Programmed cell death 1 ligand 2	Pdcd1lg2	58205	–1.8532719	0.020944852	Peng and Eckhardt, 2013
	SAM and SH3 domain containing 3	Sash3	74131	–1.00163088	0.005576389	Scheikl et al., 2009
	Marginal zone B and B1 cell-specific protein 1	Mzb1	69816	–1.854756211	7.72E-06	Flach et al., 2010
	Foxf1 adjacent non-coding developmental regulatory RNA	FOXF1	68790	1.028879446	0.042391573	NCBI
	Anoctamin 9	ANO9	71345	1.104812889	0.04292976	Rock et al., 2008
	Tripartite motif-containing 6	TRIM6	94088	1.104616713	0.043674764	Sato et al., 2012
Related to lung development or development	Matrix metallopeptidase 27	MMP 27	234911	2.088654169	0.041785907	Nuttall et al., 2004
	Erythropoietin receptor	Epor	13857	1.263048651	0.000502759	NCBI
	GATA binding protein 1	Gata 1	14460	1.821382745	0.04829584	NCBI
	Serine (or cysteine) peptidase inhibitor, clade A (alpha-1 antiproteinase, antitrypsin), member 7	Serpina7	331535	2.231550156	0.028961618	NCBI
Related to cell morphology or membrane integrity	Villin	Villin	22349	1.077160094	0.026732286	Khurana and George, 2008
	Crumbs family member 1, photoreceptor morphogenesis associated	CRB1	170788	2.51928641	0.008667091	Mehalow et al., 2003

## Discussion

The eradication of helminths (and other pathogens) is suggested to have resulted in decreased immune-regulatory ability, which might be the cause of the increasing prevalence of allergic and autoimmune disorders especially in developed and urbanized countries (de Ruiter et al., 2017; Harnett and Harnett, 2017; Bach, 2018). The protective effect of parasitic infection against allergies and autoimmune disease has been extensively explored especially after the hygiene hypothesis was introduced into this field (Maizels et al., 2014), among which the immunoregulation of schistosome is best illustrated (Capron, 2011; Layland et al., 2013; Qiu et al., 2017).

In this study, to investigate how the timing of *S. japonicum* infection influenced the development of allergic asthma, we compared the protective effect of two phases of *S. japonicum* infection: lung stage and post–lung stage. We found that lung-stage *S. japonicum* infection significantly relieved OVA-induced AAI, but post–lung-stage infection showed no protective effect. Within lung-stage infection (3–7 days postinfection), schistosomula transformed from cercaria were completely located in lung tissue of the host (Rheinberg et al., 1998), which might modulate the local immune response to abate OVA-induced AAI. We postulated that this might be the reason that the protective effect of lung-stage infection was superior to post–lung-stage infection. And indeed, we found that lung-stage infection significantly upregulated Treg cell response in lung tissues.

Multiple factors such as worm species, timing, intensity and chronicity of infection, and host genetics have been investigated to illustrate the mechanisms of helminth-mediated regulation of host immunity (Cooper, 2009). Nonetheless, the relationship between helminths and asthma still remains not well understood. Mechanistic studies reported contradictory results; for example, one study showed that *S. mansoni*–mediated suppression of AAI was patency dependent and mediated by infection-induced Treg cells (Layland et al., 2013), whereas another study showed that protection mediated by *S. mansoni* egg was independent of either Treg cells or Breg cells (Obieglo et al., 2018). In the current study, we found that lung-stage *S. japonicum* infection that occurred during OVA challenge could upregulate the frequency of Treg cells and suppress OVA-specific IL-4 response. Upregulation of Treg cells by *S. japonicum* infection has been reported by few previous studies (Baru et al., 2010; Layland et al., 2013); however, to our knowledge, this is the first proof showing that the lung-stage *S. japonicum* infection can upregulate allergen OVA-specific Treg cell.

To elucidate the role of Treg cells in *S. japonicum* infection–mediated alleviation of AAI, we first analyzed the relationship between Treg cells and OVA-specific IgE and found that the frequency of Treg cells in lung negatively correlated with OVA-specific IgE. The suppression of IgE secretion by Treg cells has been observed and described by multiple previous studies (Meiler et al., 2008; Wing et al., 2008; Khan, 2020). IL-10 and CTLA-4 pathways were suggested to be associated with the suppression of IgE; however, the detailed molecular mechanism is still elusive. Furthermore, by *in vivo* depletion of Treg cell, we found that the decrease in IgE secretion was Treg cell–dependent. IgE acts as the major mediator contributing to AAI (Gabet et al., 2019). Our results demonstrated that the protective effect of *S. japonicum* infection on AAI was mediated by Treg cell–dependent inhibition of IgE, which was consistent with a previous report showing that the preventive effect of chronic *S. mansoni* infection against later AAI was also Treg cells dependent (Layland et al., 2013).

Mechanisms underlying the induction of Treg or Breg cells by helminth-related antigens have been reported (Zaccone et al., 2009; Haeberlein et al., 2017). However, we did not find out the exact active molecules of schistosome that led to the upregulation of Treg cells in this study. Nonetheless, we think that it is very likely that the observed protective effect was a collective result of multiple components of the schistosome, as previous studies showed that multiple enzymes released by schistosomula could regulate host immunity (Hansell et al., 2008; Liu et al., 2015). We plan to acutely define these components in the future.

Instead of identifying effector antigens, in this study, we tried to understand how the lung-stage *S. japonicum* infection influences local immune responses in lung. To do so, we performed a transcriptomic comparison between lung tissues of schistosome-infected and non-infected mice. The results showed that, after lung-stage *S. japonicum* infection, most genes related to immune response were downregulated (70/84), which implied that the general immune state in lung tended to be downregulated by *S. japonicum* infection. Among these genes, we found that three genes (CD46, Epor, and Klra17) reported to promote Treg cell response were upregulated, and eight genes (Clec7a, CCR6, Spi-B, ABCG1, ADA, Ctsk, Ctss, and Ptgir) reported to inhibit Treg cell response were downregulated in schistosome-infected mice, suggesting that *S. japonicum* infection generated a milieu facilitating Treg cell induction in the lung. In the meantime, we also observed that some molecules reported to facilitate the function of B cells or plasma cells were downregulated, which was consistent with our finding that IgE response was suppressed.

Collectively, our study showed that lung-stage *S. japonicum* infection established a regulatory environment in the lungs, which can help to relieve OVA-induced AAI in a mouse model. Although the exact mechanism of Treg cell upregulation remains elusive, our data showed that lung-stage *S. japonicum* infection can improve the population of allergen-specific Treg cells that suppress IgE production. These results highlight the value of lung-stage *S. japonicum* infection as a potential therapy for allergic asthma.

## Data Availability Statement

RNA sequencing data are deposited in the SRA database SRA, SRA accession number: PRJNA609083.

## Ethics Statement

The animal study was reviewed and approved by Laboratory Animal Welfare & Ethic Committee (LAWEC) of National Institute of Parasitic Diseases (Permit Number: IPD-2016-7).

## Author Contributions

ZL, WZ, WY, BZ, YX, XW, YW, QW, CS, and JL conducted the experiment. ZL, YW, and WH designed the experiment and analyzed the data. FL analyzed the data of RNA-sequence. ZW, FQ, JC, and BX provided intellectual input and aided the experimental design. ZL wrote the manuscript. YW and WH revised the manuscript. All the authors contributed to the article and approved the submitted version.

## Conflict of Interest

The authors declare that the research was conducted in the absence of any commercial or financial relationships that could be construed as a potential conflict of interest.

## Footnotes

1 http://www.bioinformatics.babraham.ac.uk/projects/fastqc/

2 http://www.usadellab.org/cms/index.php?page=trimmomatic

3 http://pantherdb.org/

## Supplementary Material

Supplementary Figure 1 Comparisons of concentrations of IL-13, IL-10, IL-17A, and IFN-γ in BALF. FI indicated fluorescence intensity. Data are shown as mean ± SEM. *n* = 5 or 6 mice per group per experiment, experiment performed twice. **P* < 0.05, ***P* < 0.01, ****P* < 0.001 by one-way analysis of variance (ANOVA) with Tukey test.

Supplementary Figure 2 The influence of lung-stage *S. japonicum* infection on OVA-specific IFN-γ and IL-4 response after OVA challenge. **(A)** Gating strategy of flow cytometry. **(B)** Frequencies of OVA-specific CD3^+^CD4^+^IL-4^+^ T cells, CD3^+^CD4^+^IFN-γ^+^ T cells, and their ratios in lung and LDLN. Data are shown as mean ± SEM, *n* = 8 mice per group per experiment, experiment performed twice. **P* < 0.05, ***P* < 0.01 and NS, not significant by one-way analysis of variance (ANOVA) with Tukey test.

Supplementary Figure 3 The efficiency of Treg cell deletion. The representative flow cytometry of CD25^+^Foxp3^+^ Treg cells in peripheral blood treated with anti-CD25 neutralized antibody.

Supplementary Figure 4 Panther pathway analysis of DEGs between lung-stage *S. japonicum*–infected mice and no-treatment control mice after OVA challenge.
