# Supplementary figures and images for: Allergen-Specific Treg Cells Upregulated by Lung-Stage S. japonicum Infection Alleviates Allergic Airway Inflammation
Source: Front Cell Dev Biol. 2021 Jun 8;9:678377. doi: 10.3389/fcell.2021.678377 (PMC8217774; doi:10.3389/fcell.2021.678377)

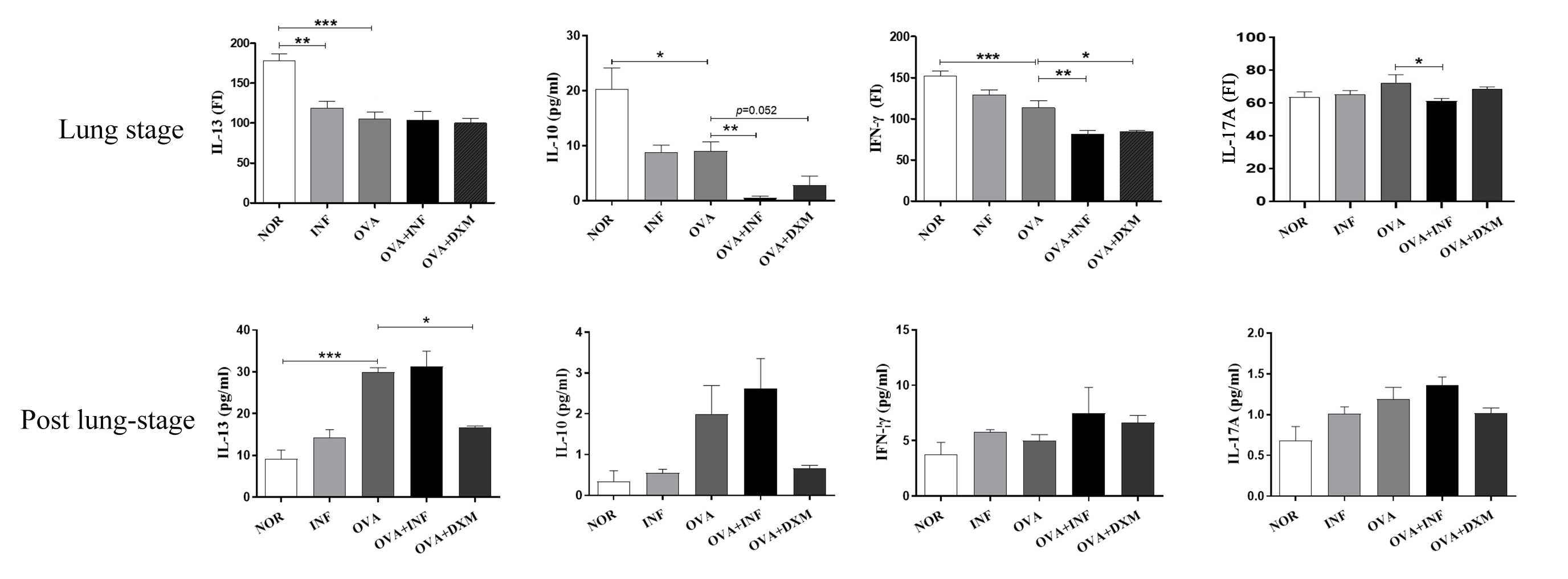

Supplement: Supplementary Figure 1 — Comparisons of concentrations of IL-13, IL-10, IL-17A, and IFN-γ in BALF. FI indicated fluorescence intensity. Data are shown as mean ± SEM. n = 5 or 6 mice per group per experiment, experiment performed twice. *P < 0.05, **P < 0.01, ***P < 0.001 by one-way analysis of variance (ANOVA) with Tukey test. [file Image_1.TIF]

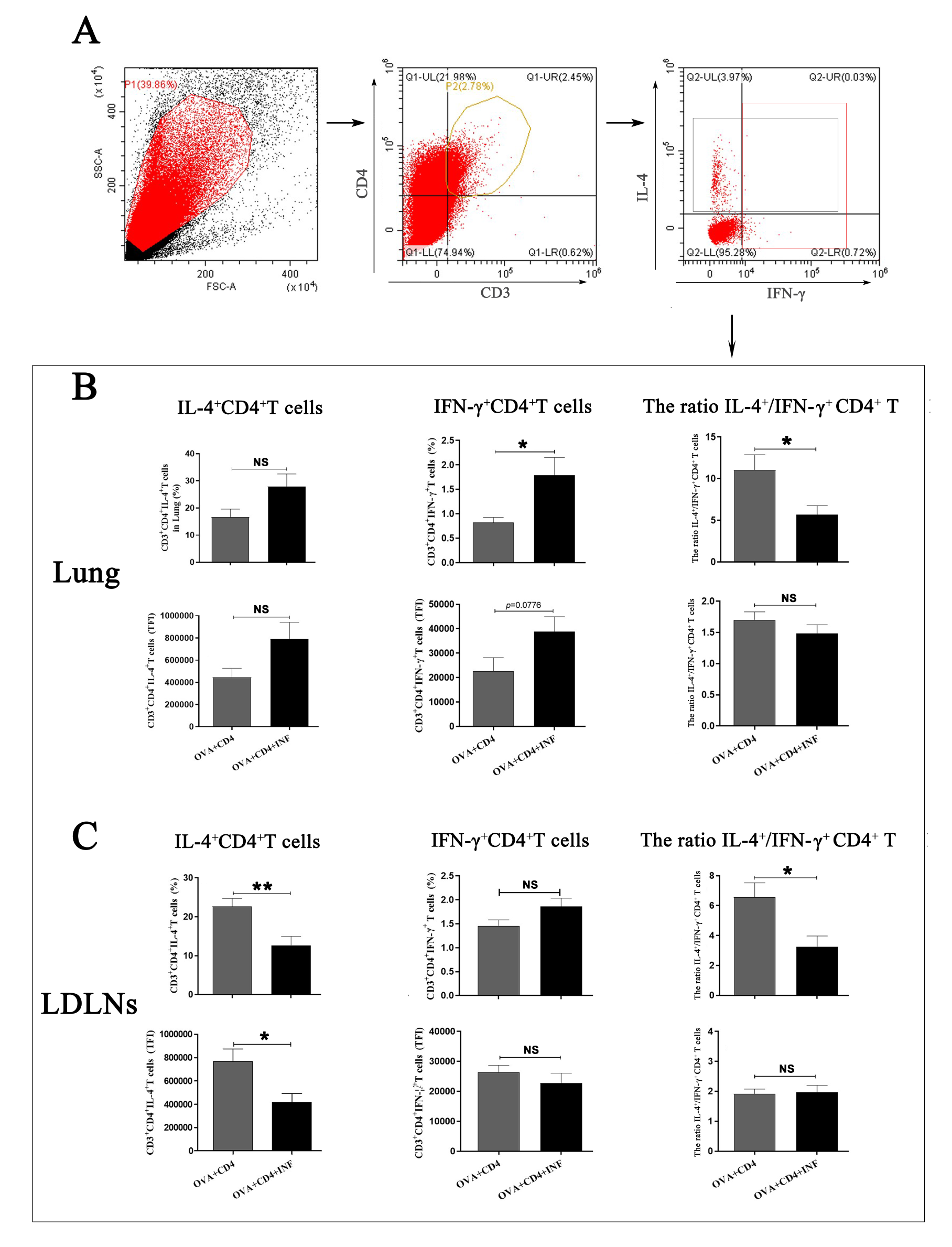

Supplement: Supplementary Figure 2 — The influence of lung-stage S. japonicum infection on OVA-specific IFN-γ and IL-4 response after OVA challenge. (A) Gating strategy of flow cytometry. (B) Frequencies of OVA-specific CD3+CD4+IL-4+ T cells, CD3+CD4+IFN-γ+ T cells, and their ratios in lung and LDLN. Data are shown as mean ± SEM, n = 8 mice per group per experiment, experiment performed twice. *P < 0.05, **P < 0.01 and NS, not significant by one-way analysis of variance (ANOVA) with Tukey test. [file Image_2.TIF]

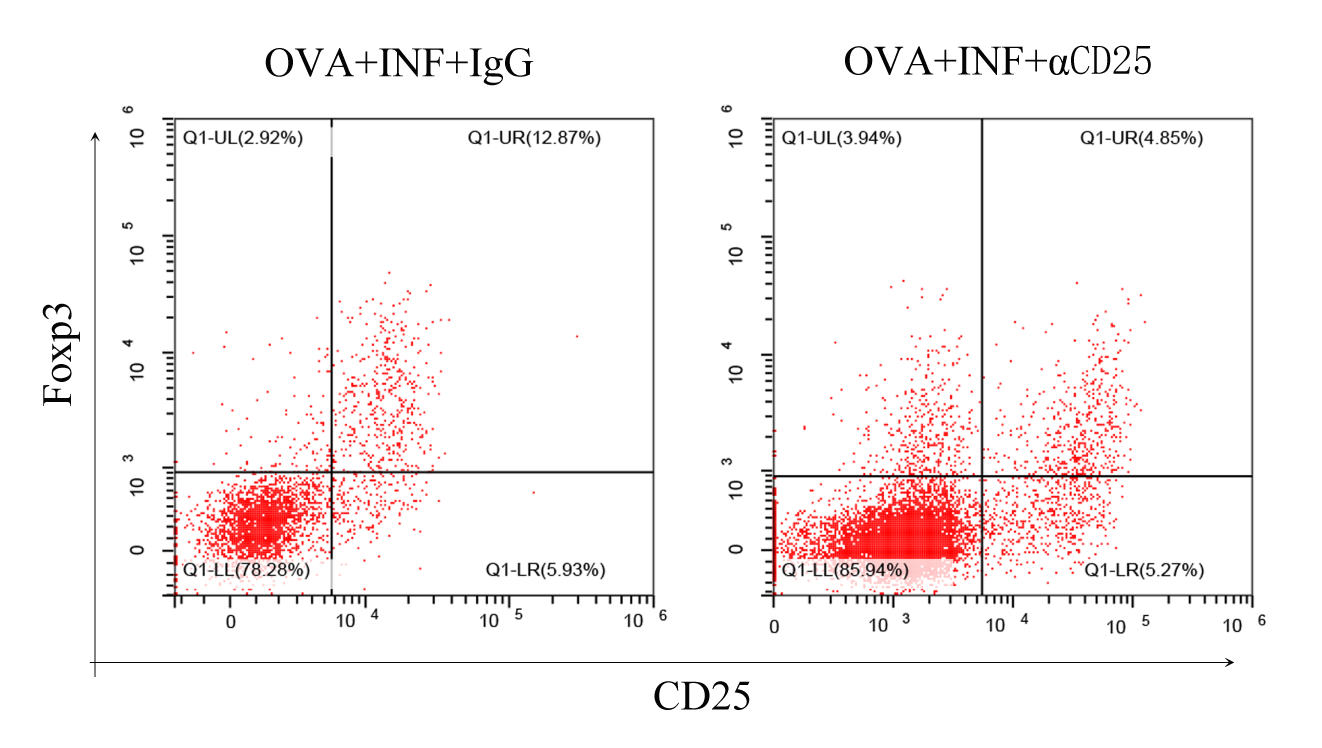

Supplement: Supplementary Figure 3 — The efficiency of Treg cell deletion. The representative flow cytometry of CD25+Foxp3+ Treg cells in peripheral blood treated with anti-CD25 neutralized antibody. [file Image_3.TIF]

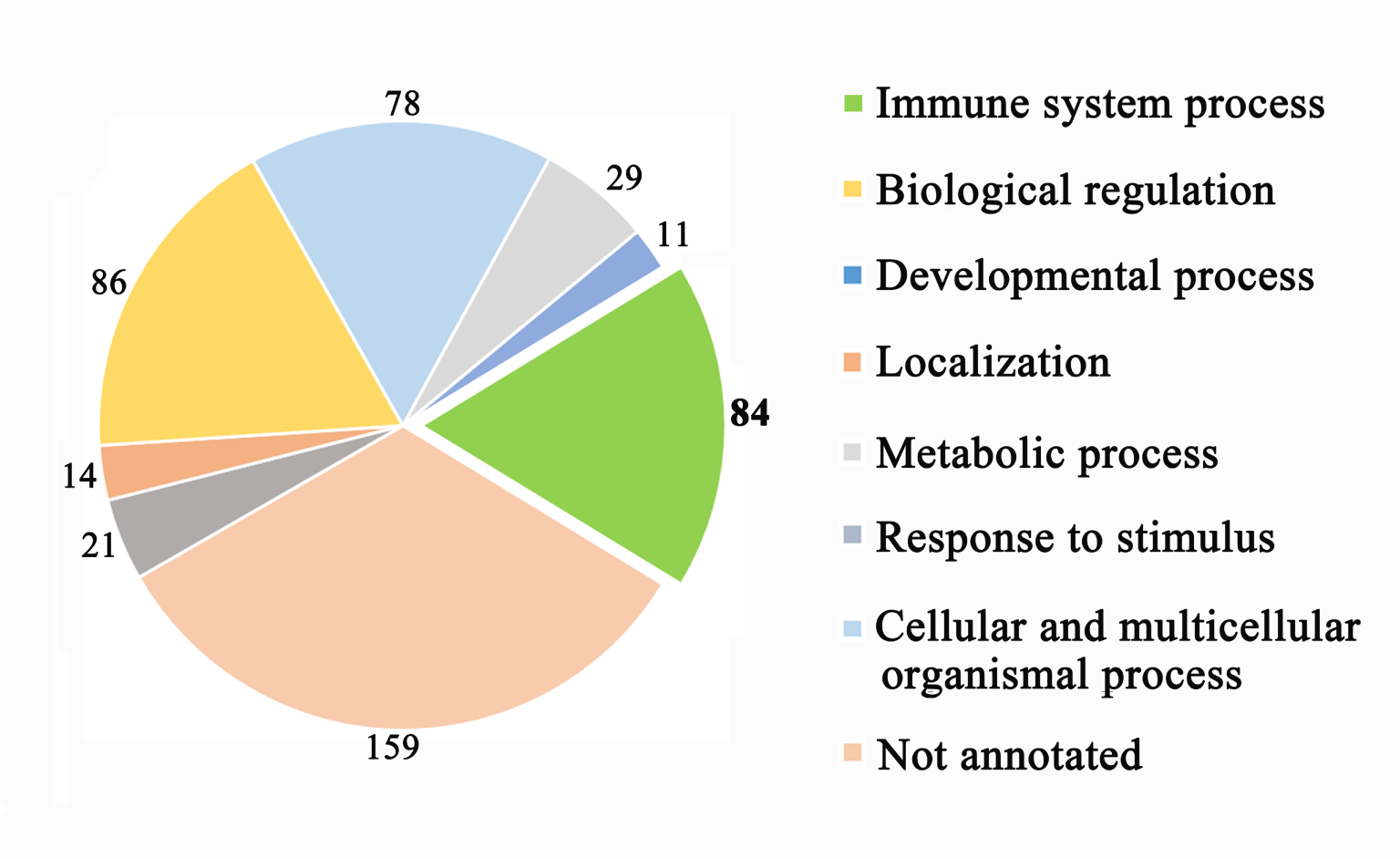

Supplement: Supplementary Figure 4 — Panther pathway analysis of DEGs between lung-stage S. japonicum–infected mice and no-treatment control mice after OVA challenge. [file Image_4.TIF]
